# The impact of age-related hearing loss on structural neuroanatomy: A meta-analysis

**DOI:** 10.3389/fneur.2022.950997

**Published:** 2022-08-08

**Authors:** Kate Slade, Johannes H. Reilly, Kamila Jablonska, El Smith, Lawrence D. Hayes, Christopher J. Plack, Helen E. Nuttall

**Affiliations:** ^1^Department of Psychology, Faculty of Science and Technology, Lancaster University, Lancaster, United Kingdom; ^2^School of Health and Life Sciences, Sport and Physical Activity Research Institute, University of the West of Scotland, Glasgow, United Kingdom; ^3^Manchester Centre for Audiology and Deafness, School of Health Sciences, University of Manchester, Manchester, United Kingdom

**Keywords:** age-related hearing loss (ARHL), gray matter (GM), structural MRI, brain volume, hearing loss, meta-analysis

## Abstract

This meta-analysis investigated the association between age-related hearing loss and structural neuroanatomy, specifically changes to gray matter volume. Hearing loss is associated with increased risk of cognitive decline. Hence, understanding the effects of hearing loss in older age on brain health is essential. We reviewed studies which compared older participants with hearing loss (age-related hearing loss: ARHL) to older adults without clinical hearing loss (no-ARHL), on neuroanatomical outcomes, specifically gray matter (GM) volume as measured by magnetic resonance imaging. A total of five studies met the inclusion criteria, three of which were included in an analysis of whole-brain gray matter volume (ARHL group *n* = 113; no-ARHL group *n* = 138), and three were included in analyses of lobe-wise gray matter volume (ARHL group *n* = 139; no-ARHL group *n* = 162). Effect-size seed-based d mapping software was employed for whole-brain and lobe-wise analysis of gray matter volume. The analysis indicated there was no significant difference between adults with ARHL compared to those with no-ARHL in whole-brain gray matter volume. Due to lacking stereotactic coordinates, the level of gray matter in specific neuroanatomical locations could only be observed at lobe-level. These data indicate that adults with ARHL show increased gray matter atrophy in the temporal lobe only (not in occipital, parietal, or frontal), compared to adults with no-ARHL. The implications for theoretical frameworks of the hearing loss and cognitive decline relationship are discussed in relation to the results. This meta-analysis was pre-registered on PROSPERO (CRD42021265375).

**Systematic Review Registration:** https://www.crd.york.ac.uk/prospero/display_record.php?RecordID=265375, PROSPERO CRD42021265375.

## Introduction

The population is aging, meaning that health issues which affect older adults become more prevalent (1), impacting on the older population's quality of life and placing increasing pressure on health care services. Two such health concerns are hearing loss and dementia. In the UK around 70% of people aged 70+ experience hearing loss (2), and around 7.1% of over 65's, rising to 14% of those over 80, are living with dementia (3). Critically, there is evidence that these health concerns may be associated. Hearing loss has been identified as the largest potentially modifiable risk factor for dementia (4). Hearing loss could account for as much as 8% of global dementia cases (5). It is likely that many risk factors are associated, and exacerbate one another leading to increased risk of dementia in certain individuals. Considering this, understanding the neural mechanisms of hearing loss, and how they may contribute to the association between hearing loss and dementia, is a priority.

Age-related hearing loss (ARHL) is often caused by degeneration of the inner and outer hair cells within the cochlea. These cells are responsible for the transduction of sound, and their atrophy can manifest in high-frequency hearing loss (6). Age-related atrophies in the peripheral auditory system can also be observed in the stria vascularis, a cochlea structure responsible for maintaining metabolic processes (7), or in degeneration of spiral ganglion cells, the initial neurons in the pathway from the ear to the brain (8). Importantly, evidence suggests that changes and atrophies in people with ARHL do not end at the peripheral auditory system, but are also evident in the auditory pathway and auditory cortex (9, 10). Understanding the cortical changes, in auditory areas and beyond, would provide valuable insights into how the brain changes in people with ARHL, and provide evidence for the mechanisms that underpin the association between hearing loss and cognitive decline.

A number of potential explanations for the relation between hearing loss and dementia have been proposed. These include non-causal hypotheses such as: (1) The common cause hypothesis, which suggests that rather than hearing loss leading to dementia, there is a common neuro-degenerative pathology which underlies both conditions such as general aging or vascular disease (11, 12); or (2) The hearing bias in cognitive assessment hypothesis, which suggests that there may be an overestimation of the link between hearing loss and dementia, because untreated hearing loss could be a significant confound in clinical cognitive assessments that rely on auditory presentation (13, 14). However, the relation between hearing loss and cognitive decline remains after controlling for age and vascular factors (15, 16), and hearing loss has been found to be associated with poorer cognitive functioning even when the cognitive assessments do not rely on verbal communication (17). As such, a causal mechanism may be more likely. Causal hypotheses include: (1) The cognitive load (or information degradation) hypothesis, which theorizes that people with ARHL are required to use more cognitive resources for speech perception leaving fewer available for general cognitive processes, which could lead to the symptoms of dementia (18); and (2) The sensory deprivation hypothesis, which postulates that reduced sensory input from the ear leads to reduced neural activation, cortical re-organization, and atrophy across brain areas involved with speech perception (10, 19). Both these causal hypotheses make suggestions with regards to functional or structural neuro-cognitive changes that might accompany ARHL, including upregulation or reorganization of neural resources (20) or atrophy across speech perception networks [see (21) for a discussion of cortical changes]. As such, a comprehensive review of the current literature on the neural consequences of ARHL is required to generate evidence to refute or support these causal hypotheses.

The first step in the systematic examination of neural consequences of ARHL is to assess the evidence for tangible neuroanatomical changes, in both auditory and wider cortices. There is evidence from longitudinal studies that individuals with ARHL display accelerated gray matter (GM) atrophy in auditory cortex compared to individuals without ARHL (22), however these group differences have not always reached statistical significance (23). In cross-sectional studies, there is also evidence for decreases in whole brain volume in those with ARHL compared to those without (10), and in specific brain areas associated with speech perception including the anterior cingulate cortex (24). The mechanism by which ARHL leads to wider brain atrophy is unclear. One potential explanation is that over-reliance on wider brain networks involved in speech perception due to impaired auditory processing contributes to neural degeneration of these areas. There is evidence that individuals with ARHL, compared to those without, display increased functional connectivity across auditory and visual sensory cortices (25), and between auditory cortex and the cingulo-opercular network after controlling for both age and cognitive function (26). The over-reliance on these additional brain networks to support speech perception in individuals with ARHL could enable neural degeneration due to glutamate excitotoxicity (24). Through this mechanism, the neurons across the up-regulated brain networks may die due to prolonged activation of glutamate receptors beyond their natural capacity (27).

Despite evidence for potential up-regulation and cortical atrophy, it is still unclear as to whether or not ARHL exacerbates the cortical changes observed in aging. Heterogeneity in research methods, such as differences in participant age ranges, imaging techniques, or clinical definitions of hearing loss, as well as small sample sizes, can lead to ambiguity in interpretation of the results. This meta-analysis will deliver a systematic and specific analysis of the existing literature on neuroanatomical changes in ARHL, controlling for some of these confounds through appropriate inclusion criteria, and study quality assessment. We sought to investigate across cross-sectional and longitudinal evidence whether GM volume, as measured by MRI, differs in adults aged ≥60 years with ARHL compared to those without ARHL. In this paper, ARHL is defined by hearing thresholds above 25 dB HL for frequencies between 500 and 2,000 Hz in adults aged 60+, whereas “without ARHL” is defined by hearing thresholds below 25 dB HL for frequencies between 500 and 2,000 Hz in adults aged 60+, representing age-appropriate hearing function. It was hypothesized that we would observe (1) decreased whole brain GM volume, as well as (2) decreases in GM volume in the temporal lobe, in individuals with ARHL compared to those without ARHL.

## Methods

This meta-analysis was pre-registered on PROSPERO (PROSPERO 2021, CRD42021265375), available from: https://www.crd.york.ac.uk/prospero/display_record.php?ID=CRD42021265375) Additionally, all materials including: search strings; references obtained at all screening stages; screening manuals and inter-rater consistency data; extracted data; and analysis files can be found in the project's repository on the Open Science Framework (OSF): https://osf.io/g5qcb/.

### Literature search

An initial pilot search was conducted on PubMed and Prospero according to best practice guidance (28, 29), in order to: (1) confirm whether systematic reviews and meta-analyses on this topic already existed; (2) estimate the feasibility of the meta-analysis and availability of data; and (3) identify key papers to inform the selection of appropriate key words and criteria for the final search string. Unlike the full literature search, the pilot search was characterized by iterative searching without pre-defined search strings. In-depth engagement with the literature might introduce potential bias in the construction of the full literature search. Hence, engagement with the pilot search was limited to 2 h.

Subsequently, the full literature search was conducted following PRISMA guidelines (30) and best practice guidelines from the “Cochrane Highly Sensitive Search Strategy” (31). Nine databases were searched (Table 1). To maximize effective retrieval of relevant papers, the research question was approached, inter alia, from medical (PubMed), nursing (CINAHL), and psychological (PsycINFO) perspectives. To ensure comprehensiveness, searches were not filtered in any way, e.g., by database internal filters, such as publication date, or by full-text articles (see Table 1 for exception in Scopus). If any full text could not be accessed the research team planned to contact the authors of the papers, and allow 1 week for an initial response before re-contacting. A total of 2 weeks was granted for authors to respond and provide access to papers.

**Table 1:** The databases searched and the date on which the search was conducted.

**Database**	Date of last search*
Academic Search Ultimate	05 August 2021
CINAHL	05 August 2021
Embase	05 August 2021
MEDLINE Complete	05 August 2021
PsycINFO	05 August 2021
PubMed	05 August 2021
Scopus	10 August 2021^†^
The Cochrane Library	05 August 2021
Web of Science	05 August 2021

*Articles published after termination of each search on the respective date were not included. ^†^The final search in Scopus was delayed relative to other databases, as the Scopus search initially retrieved over 30,000 articles. The search was optimized with advice sought from a librarian. Specifically, search terms of tangential relevance, e.g., cognition, that were included in the searches of other databases were omitted in the search of Scopus. Additionally, filters were applied to limit the search to articles in the English language published after 1980, when MRI was increasingly used clinically.

Search strings (see Supplementary materials 1 for full details) were constructed using keywords, free-text terms, and search functions (Boolean operators, near searches, truncation, wild card symbols, quotations), to ensure specificity and sensitivity across databases. An example of the search terms included: “hearing loss” or “hearing impairment” or “presbycusis,” and “voxel-based” or “morphometry” or “magnetic resonance imaging” or “cortical thickness” or “gray matter,” and “older adult.” The final search strings, selected keywords, and Boolean operators were reviewed by a librarian to ensure adherence to best practice insights. To manage resource and time constraints, the search was limited to titles and abstracts.

Prior to conducting the literature search, a strategy test of sensitivity was completed in which four key papers that satisfied the inclusion criteria were identified using a database not used in the final search to avoid bias (Google Scholar). The sensitivity of the search strings was evaluated by testing how many of these four key papers indexed in the selected databases (Table 1) could be retrieved. Once the search sensitivity was acceptable, the literature search was conducted across the selected databases. All key papers were retrieved with the search indicating high likelihood that the search would successfully identify relevant articles.

### Article screening

**Figure 1:**
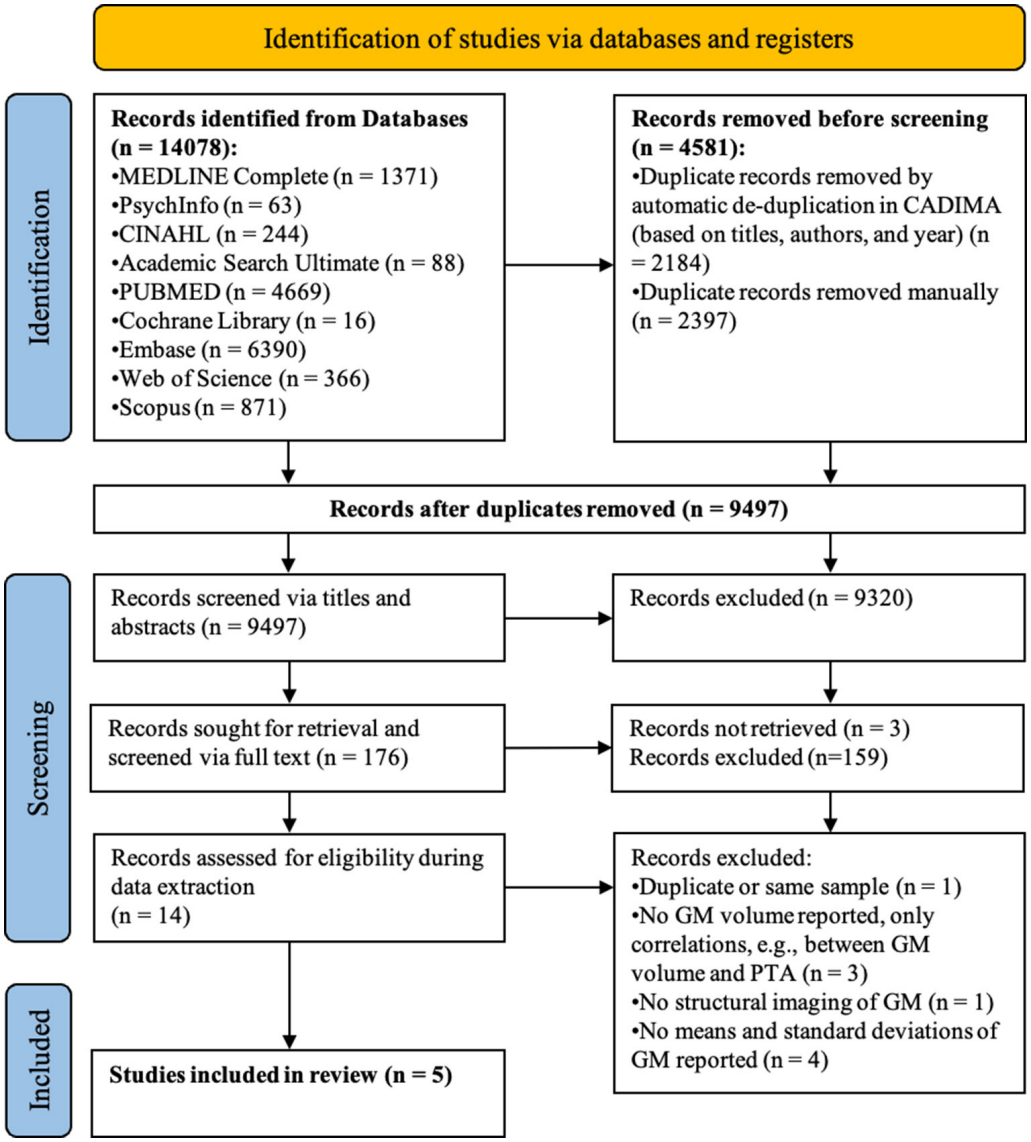
PRISMA flow diagram detailing the number of articles selected at each review stage. GM refers to gray matter.

Articles (*n* = 14,078) retrieved from the literature search were imported to the reference manager software CADIMA [https://www.cadima.info/; for a review, see (32)]. An overview of the articles retained at each stage of the screening process can be found in the PRISMA flow diagram presented in Figure 1 (33). Any duplicated articles, retrieved by more than one database, were removed in a two-step process: (1) automatic de-duplication based on congruity in authors, title, and year of publication; and (2) manual de-duplication by two raters based on abstracts.

Unique articles (*n* = 9,497) were screened by four raters for inclusion according to specific criteria (see the associated OSF repository for the full criteria used: https://osf.io/g5qcb/) in two consecutive stages: (1) title-abstract; and (2) full-text screening. Before each screening stage, the consistency between raters was checked with inter-rater reliability tests (Table 2). A subset of articles (60 at title-abstract screening stage, and 40 at full-text screening stage) were screened by two raters in parallel until at least 80% agreement was reached for each criterion. During screening, a manual with the inclusion criteria, additional background information, and guidance for the use of CADIMA was provided (manuals are also available in the OSF repository).

**Table 2:** Proportional agreement of the inter-rater reliability at each screening stage.

**Criteria**	**Proportional inter-rater agreement**
Title-Abstract Screening	Overall 0.95
Criterion 1	0.10
Criterion 2	0.95
Criterion 3	0.92
Criterion 4	0.99
Criterion 5	0.90
Full-text Screening	Overall 0.87
Criterion 1	0.98
Criterion 2	0.86
Criterion 3	0.83
Criterion 4	0.80

After consistency checks, 10% of all titles and abstracts and 30% of full-texts were double screened by two independent raters in parallel. During this initial period in screening, inconsistencies were resolved through group discussion and if necessary, information was added to the screening manual to clarify eligibility criteria. Training and extensive guidance was provided to ensure all raters fully understood the application of eligibility criteria before the remaining articles were independently screened. Throughout this process, raters met weekly to resolve outstanding questions. Raters were instructed to include rather than exclude articles if unsure, to prevent false exclusion of papers.

The final set of articles that passed title-abstract (*n* = 176) and full-text screening stages (*n* = 14), were checked for listing in the Retraction Watch Database (http://retractiondatabase.org/) to ensure that only studies not retracted were included.

Articles were screened for inclusion along a set of pre-defined eligibility criteria for (1) the title-abstract and (2) the full-text screening stages. These criteria were designed in line with the PICO/PECO framework (34, 35), which clarifies the review objectives and inclusion criteria across four domains: Population (P), Intervention/Exposure (I/E), Comparator (C), and Outcomes (O). To meet the inclusion criteria, articles were required to be original research, containing empirical data, and provided in English. Additionally, the articles needed to meet the following PICO/PECO criteria. (P) it was required that participants be older adults (average age of 60+ at the time of at least one study session) without clinical psychological or neurological illnesses, who either had or did not have age-related hearing loss (ARHL). (I/E) ARHL was defined as a pure tone average (PTA) of >25 dB HL across 0.5–2 kHz and no-ARHL was defined as a PTA of ≤ 25 dB HL averaged across 0.5–2 kHz (36, 37). (C) Studies needed to compare at least two groups, one group with ARHL and one group with no-ARHL, either longitudinally or cross-sectionally. (O) Outcome measures needed to include voxel-based morphometry data (VBM) as measured by magnetic resonance imaging (MRI) available for both groups. The outcome measures of interest were gray matter (GM) volumes for specific brain regions or for the whole brain.

### Data extraction

Data extraction was performed manually by four reviewers with identically structured Microsoft Excel (2018) forms. For each study, data were extracted by at least two independent reviewers and then checked for agreement, to decrease the possibility of manual errors (38). Any inconsistencies in the extracted data were resolved through discussion. A data extraction manual was provided (available in the OSF repository). Where data were presented visually only, means and standard deviations were read from graphs. If non-significance or significance was reported without associated exact *p*-values, the *p*-value was assumed to be *p* = 0.05 and *p* = 0.04, respectively [based on Anatürk et al. (38)]. The main source of heterogeneity in analysis is likely to stem from sample characteristics, study design, and imaging technique. Therefore, data extraction included participant demographics for both ARHL and no-ARHL groups (sample size, age, sex, PTA), study design (timeframe, sampling method, timescale of longitudinal measurements), details of image acquisition (MRI field strength, smoothing kernel, slice thickness, voxel size, mask, normalization space), and outcome measures (e.g., (un)corrected *p*-values, effect sizes, mean and standard deviation of whole-brain and regional GM volumetric measurements). Any papers found not to meet the inclusion criteria at data extraction stage were excluded (for details, see the PRISMA flowchart, Figure 1, and materials on the OSF).

Nine papers were excluded at this stage due to the nature of the reported data or ineligibility. One was a duplicate reference. The reasons for exclusion and main findings of the remaining eight papers are reported here. Three papers (39–41) reported only correlational or regression data; due to the nature of the statistical methodology, these papers did not meet the inclusion criteria of specific group comparison between no-ARHL and ARHL groups. Across two of these studies, authors reported that ARHL only had a small effect on: GM volume in Hershel's gyrus (41); and cortical thinning in the right superior temporal and left dorsolateral frontal areas, in women only with right ear hearing loss (39). The third study reported correlations between brain volume changes and functional impairment factors within ARHL groups only (40). Another paper was ineligible as only data on white matter were reported, for which there were no differences between ARHL and no-ARHL groups (42). Finally, four papers that did not report means, standard deviations, or statistical values that could be employed in this meta-analysis were excluded due to lack of data provision following the procedure for author contact mentioned in section 2.1. Two of these papers reported no significant differences in brain volume between ARHL and no-ARHL groups (43, 44). Another reported significant differences in brain volume and thickness across temporal lobe regions, and areas of the cingulate cortex, in ARHL compared to no-ARHL (45), whereas another reported reduced GM volume in the middle frontal gyrus, but not in auditory regions, in ARHL compared to no-ARHL (46).

The remaining papers (*n* = 5) were included in this meta-analysis.

### Critical appraisal

A framework to appraise critically the quality of the studies included in this meta-analysis was created using previously established appraisal tools. These tools typically comprise a set of questions that raters use to evaluate the research methodologies of included studies. Such frameworks allow appraisal of study quality and risk of bias, to evaluate the reliability and validity of studies' findings, and whether findings are representative and generalizable at population-level. No automation tools were used in this process. The critical appraisal tool was based on an adapted version of the trialed AXIS appraisal framework (47) and response options were based on QualSyst (48). Individual criteria of the original AXIS tool were omitted or included based on Müller et al. (28), the STROBE statement (49), and GRADE (50, 51). To minimize subjectivity (28), each paper included in the analysis was appraised by two raters independently, and disagreements resolved by discussion or ultimately, a third rater. Raters were trained and received an explanatory manual. To assess homogeneity in methods and outcomes across studies, the critical appraisal accounted for whether or not research controlled for confounding factors (e.g., sex, education, smoking status, age), as well as methodological factors that could influence data interpretation (e.g., sample size). The appraisal manual and method of calculation are available in the OSF repository.

### Statistical analysis

Statistical analysis was conducted using effect-size seed-based d mapping (ES-SDM) software to perform a random-effects meta-analysis (52), the software was developed to aid the meta-analysis of voxel-based data as obtained by VBM (www.sdmproject.com). VBM is a neuroimaging technique comparing GM concentration by mapping images onto a normalized stereotactic space and extracting GM volumes, smoothing data, and finally, comparing group GM volume differences via voxel-wise comparison (53). ES-SDM is described in detail elsewhere (52, 54) and has previously been tested for reliability (55, 56), including for GM volume comparison (57). ES-SDM calculates Hedges' *g* effect sizes for mean analysis, based on group means, and standard deviations (54). Hedges' *g* uses a pooled and weighted standard deviation based on sample size and is thus more accurate for small sample sizes (<20) than Cohen's *d* which uses a normal standard deviation (58–60). The inclusion of non-significant findings in the analysis addresses bias toward significant overall results.

The analysis of GM volumes in ES-SDM was a mean analysis providing Hedges' *g* and corresponding *z*- and *p*-values, as well as standard error, the lower and upper bounds of the effect size for each study, and a mean across studies. *Q* statistics were used to assess inter-study heterogeneity of effect sizes. The analysis followed the ES-SDM manual (available here www.sdmproject.com). Furthermore, we verified the analysis in RStudio [R version 4.1.0, (61)], using the *metafor()* package to conduct a random-effects model meta-analysis (62), and to produce the associated forest and funnel plots. The data analysis obtained was the same in ES-SDM and R. The R code is provided in the OSF repository: https://osf.io/g5qcb.

#### Calculation of missing standard deviations

Under the assumption that data were normally distributed, missing standard deviations were calculated from confidence intervals using the following formula (30):


(1) $$SD=\sqrt{N}\;x\frac{upper\;limit-lower\;limit}{3.92}$$

#### ES-SDM and R analysis

Sample sizes of both groups (ARHL vs. no-ARHL), means and standard deviations were entered for each study and each region of interest (ROI) into ES-SDM. Separate analyses were conducted for whole-brain and lobe-wise GM volume using the same ES-SDM “globals” calculator as it relies on mean analysis and is, therefore, also suitable for analysis of mean ROI data. To compare ROIs, ROIs were collated into frontal, temporal, parietal, and occipital lobes. The collation was completed following the papers' verbal labels of ROIs (e.g., superior temporal lobe was allocated to the temporal cortex) and widely accepted localisations, e.g., precentral gyrus is undisputedly considered to lie in the frontal lobe. If allocation to a lobe was unclear, a neuroanatomy textbook was consulted (63). The same data were entered into R and separate meta-analyses were conducted for whole-brain, frontal, temporal, parietal, and occipital lobe data, as was done in ES-SDM software.

## Results

Of the 9,497 articles screened, five satisfied all inclusion criteria (see Figure 1 for the PRISMA flow diagram). During title-abstract screening, a total of 413 inter-rater inconsistencies were resolved of which only 102 affected inclusion (*n* = 25) or exclusion (*n* = 77) of the article. During full-text screening there were 37 inconsistencies of which 16 affected inclusion (*n* = 2) or exclusion (*n* = 14). The number of articles that were at first included, but through discussion of inconsistencies excluded, can be explained by the instructions to screeners to be more lenient than conservative in case of uncertainty when judging whether or not the articles fulfilled screening criteria.

Across both screening stages, the criteria that caused most inconsistencies were whether or not participants were at least 60 years old and (neurologically) healthy, as well as whether or not the study made a direct comparison of neuroanatomical differences between groups.

### Heterogeneity of effect sizes and evaluation of study quality

Descriptive statistics of the meta-analyzed studies are presented in Tables 3, 4. Only one of the five included studies adopted a longitudinal approach. As such it was not possible to meta-analyze rate-of-change in GM volume over time. Therefore, all included effects reflect cross-sectional comparisons between participant groups with and without ARHL, regardless of longitudinal or cross-sectional study design.

**Table 3:** Means, and standard deviations where applicable, of the data extracted for papers used in the whole-brain analysis.

**Study**	**Design**	**Sample size (M/F)**		**Age (SD)**		**PTA (SD)**		**NH PTA definition**	**MRI field strength (T)**	**Critical appraisal score**
		**HL**	**NH**	**HL**	**NH**	**HL**	**NH**			
Chen et al. (64)	CS	22 (10/12)	23 (11/12)	63.59 (2.38)	64.74 (2.65)	34.54 (4.63)	14.82 (1.73)	≤ 25 dB at 0.25–8 kHz	1	0.83
Lin et al. (22)	L	51 (40/11)	75 (36/39)	73.80 (7.30)	67 (6.90)	N/A	N/A	≤ 25 dB at 0.5–4 kHz	1.5	0.88
Xing et al. (65)	CS	40 (19/21)	40 (18/22)	63.60 (7.07)	61.55 (3.72)	32.69 (3.87)	16.17 (2.22)	≤ 25 dB at 0.25–8 kHz	1	0.88

*Chen et al. (64) ensured participant groups were matched on age, sex, and education, and also found minimal group differences across cognitive performance domains. Xing et al. (65) also ensured matched groups on age, sex, and education, and included these factors as covariates in analyses. Further, groups displayed statistically similar cognitive functioning across the majority of tests. Lin et al. (22) controlled for intracranial volume, smoking, interactions between, time, HL, age, and sex. CS refers to cross-sectional and L refers to longitudinal*.

**Table 4:** Means, and standard deviations where applicable, of the data extracted for papers used in the lobe-wise analyses.

**Study**	**Design**	**Sample size (M/F)**		**Age (SD)**		**PTA (SD)**		**NH PTA definition**	**MRI field strength (T)**	**Critical appraisal score**
		**HL**	**NH**	**HL**	**NH**	**HL**	**NH**			
Belkhiria et al. (66)	CS	55 (23/32)	56 (19/37)	75.38 (5.20)	72.53 (5.41)	36.27 (9.50)	17.08 (4.80)	<25 dB at 0.5–4 kHz	3	0.83
Belkhiria et al. (24)	CS	33 (12/11)	31 (6/25)	73.78 (5.79)	70.84 (4.84)	25.68 (4.86)	14.16 (3.15)	≤ 0 dB at 0.5–4 kHz	1	0.89
Lin et al. (22)	L	51 (40/11)	75 (36/39)	73.80 (7.30)	67 (6.90)	N/A	N/A	≤ 25 dB at 0.5–4 kHz	1.5	0.88

*Belkhiria et al. (66) controlled for education, cognitive abilities, visuospatial capacities, (neuro-) psychiatric symptoms. Belkhiria et al. (24) controlled for education, cognitive abilities, dementia, smoking, cardiovascular risk factors, diabetes, depression. Lin et al. (22) controlled for intracranial volume, hypertension, smoking, interactions between time, HL, age and sex. CS refers to cross-sectional and L refers to longitudinal*.

Critical appraisal of the included studies was conducted by a minimum of two raters to assess research quality and risk of bias to evaluate. Studies were assessed across a range of criteria, including whether or not research controlled for important confounding factors that could influence hearing status or brain structure (e.g., sex, age, education). In one study, it was unclear whether confounding variables were controlled for in analyses, but the two groups (ARHL and no-ARHL) were matched for age, sex, and education, and showed statistically similar cognitive functioning across a range of tests (64). Critically, the four remaining studies state explicitly that statistical analyses accounted for both age and sex (22, 24, 65, 66). Further, three studies controlled for education (24, 65, 66), and three controlled for total estimated intracranial volume (22, 24, 65). In the one longitudinal study included here, the additional variables of hypertension, smoking, hearing impairment, and years since baseline, were included as covariates in analyses (22). Importantly, across the studies included in this meta-analysis, all considered the impact of key confounding variables (e.g., age) on the analyzes, allowing for clearer interpretation of the relation between ARHL and GM volume.

Overall, critical appraisal scores did not lie below 0.83, indicating high methodological quality (48). In combination with the observation that all ratings fell between 0.83 and 0.89, it is unlikely that methodological inadequacies skewed results or studies formed subgroups of studies with high and low methodological quality. However, it should be noted that a source of bias might be the consistently partial fulfillment of a sampling process likely to represent the target population. All studies employed convenience sampling (recruiting from hospital settings or previous study cohorts) and acknowledged this as a limitation. The results in this meta-analysis are consequently subject to the same constraints in generalizability of results.

Across analyses, the *Q* statistic did not reach significance indicating no significant heterogeneity of effect sizes between studies (whole-brain, *Q*(2) = 3.93, *p* = 0.14; frontal lobe, *Q*(7) = 3.11, *p* = 0.87; temporal lobe, *Q*(26) = 17.59, *p* = 0.67; parietal lobe *Q*(2) = 2.78, *p* = 0.10; occipital lobe, *Q*(4) = 2.43, *p* = 0.66). This homogeneity, in combination with the results from the critical appraisal, suggest no significant variation in the studies' characteristics and that the heterogeneity likely stems from sampling error alone. Thus, it is unlikely that underlying variation in methodology or participant groups between studies skewed the results (67).

### Whole-brain and lobe-wise analysis

**Figure 2:**
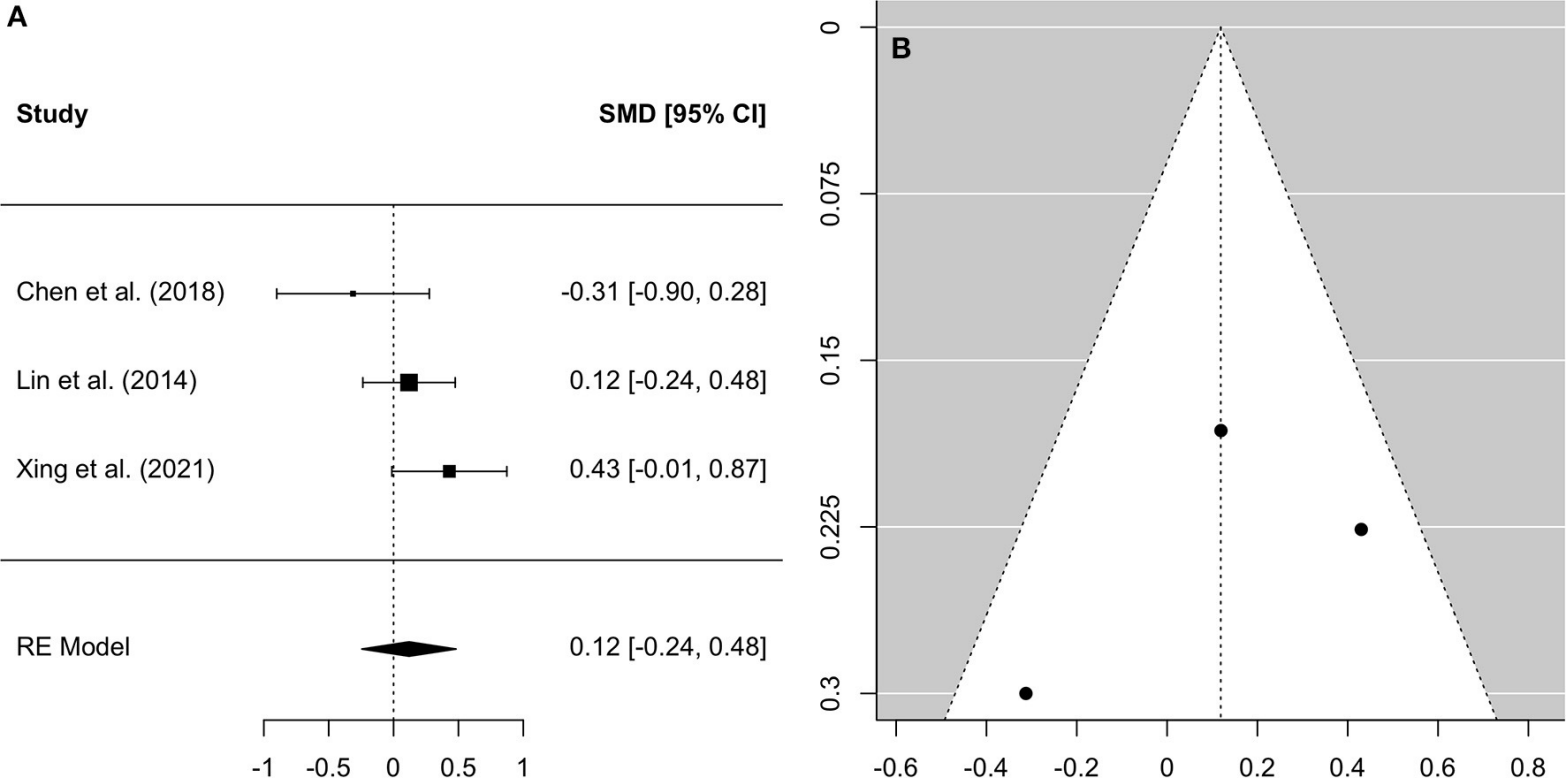
Forest plot **(A)** and funnel plot **(B)** of the whole-brain Analysis. In the forest plot **(A)** negative values on the x-axis indicate gray matter atrophy.

**Figure 3:**
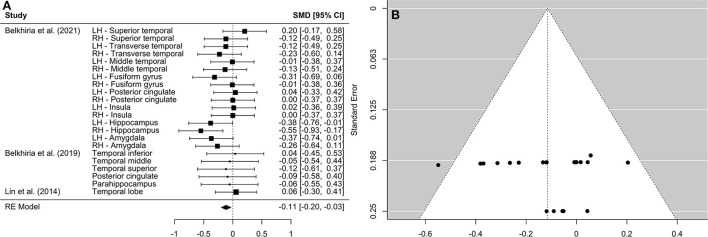
Forest plot **(A)** and funnel plot **(B)** of the temporal lobe analysis. In the forest plot **(A)** negative values on the x-axis indicate gray matter atrophy.

**Figure 4:**
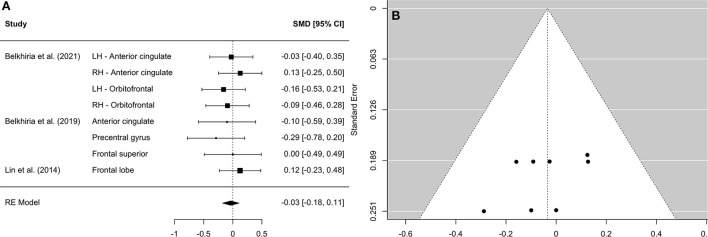
Forest plot **(A)** and funnel plot **(B)** of the frontal lobe analysis. In the forest plot **(A)** negative values on the x-axis indicate gray matter atrophy.

**Figure 5:**
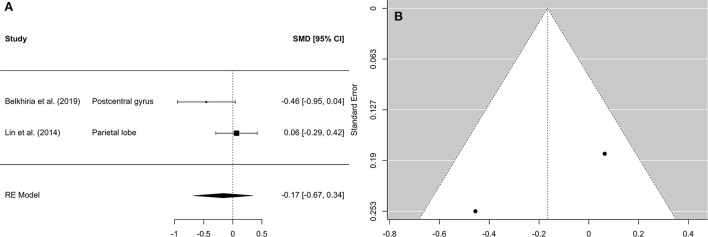
Forest plot **(A)** and funnel plot **(B)** of the parietal lobe analysis. In the forest plot **(A)** negative values on the x-axis indicate gray matter atrophy.

**Figure 6:**
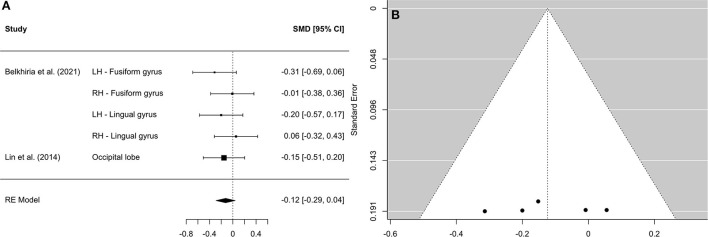
Forest plot **(A)** and funnel plot **(B)** of the occipital lobe analysis. In the forest plot **(A)** negative values on the x-axis indicate gray matter atrophy.

A comprehensive overview and visualization of results is presented in Tables 5–8, and Figures 2–6, respectively. In comparison with group no-ARHL, group ARHL, the differences whole-brain GM volume were not significant, Hedges' *g* = 0.12, *p* = 0.52. Similar to the whole-brain GM volume, group ARHL showed lower GM volumes in lobe-wise analysis. This difference was significant in the temporal lobe (Hedges' *g* = −0.12, *p* = 0.007), but was not significant in the frontal (Hedges' *g* = −0.03, *p* = 0.64), parietal (Hedges' *g* = −0.17, *p* = 0.52), nor occipital (Hedges' *g* = −0.12, *p* = 0.14) lobes.

**Table 5:** Available means and standard deviations extracted for meta-analysis of whole-brain data.

**Study**	**Normalization space**	**GM volume (SD)**	
		**HL**	**NH**
Chen et al. (64)	MNI	564.00 (24.40)	571.20 (20.80)
Lin et al. (22)	MNI	535.10 (40.99)	530.30 (39.32)
Xing et al. (65)	MNI	32.3 (1.80)	31.6 (1.40)

**Table 6:** Available means and standard deviations extracted for meta-analysis of lobe-wise data.

**Study**	**MRI space**	**Region**	**GM volume (SD)**	
			**HL**	**NH**
**Frontal lobe**				
Belkhiria et al. (66)	TAL	LH Anterior cingulate	2.41 (0.37)	2.42 (0.39)
		RH Anterior cingulate	1.84 (0.38)	1.79 (0.40)
		LH Orbitofrontal	7.32 (0.78)	7.44 (0.72)
		RH Orbitofrontal	7.41 (0.80)	7.48 (0.72)
Belkhiria et al. (24)	TAL	Frontal superior	4.91 (0.22)	4.91 (0.18)
		Anterior cingulate	4.78 (0.33)	4.81 (0.26)
		Precentral gyrus	4.90 (0.24)	4.97 (0.24)
Lin et al. (22)	MNI	Frontal lobe	156.90 (14.76)	155.10 (14.10)
**Occipital lobe**				
Belkhiria et al. (66)	TAL	LH Fusiform gyrus	7.25 (1.09)	7.57 (0.93)
		RH Fusiform gyrus	7.35 (1.32)	7.36 (1.01)
		LH Lingual gyrus	5.77 (1.01)	5.96 (0.87)
		RH Lingual gyrus	6.10 (1.00)	6.05 (0.79)
Lin et al. (22)	MNI	Occipital lobe	74.20 (8.02)	75.40 (7.71)
**Temporal lobe**				
Belkhiria et al. (66)	TAL	LH Superior temporal	14.05 (1.71)	13.74 (1.28)
		RH Superior temporal	13.05 (1.53)	13.21 (1.13)
		LH Transverse temporal	0.90 (0.18)	0.92 (0.15)
		RH Transverse temporal	0.72 (0.13)	0.75 (0.13)
		LH Middle temporal	11.28 (1.56)	11.29 (1.44)
		RH Middle temporal	11.24 (1.53)	11.43 (1.30)
		LH Fusiform gyrus	7.25 (1.09)	7.57 (0.93)
		RH Fusiform gyrus	7.35 (1.32)	7.36 (1.01)
		LH Posterior cingulate	2.85 (0.49)	2.83 (0.40)
		RH Posterior cingulate	2.74 (0.41)	2.74 (0.48)
		LH Insula	5.42 (0.64)	5.41 (0.56)
		RH Insula	5.55 (0.63)	5.55 (0.51)
		LH Hippocampus	3.38 (0.41)	3.53 (0.37)
		RH Hippocampus	3.51 (0.45)	3.74 (0.38)
		LH Amygdala	1.32 (0.22)	1.40 (0.21)
		RH Amygdala	1.54 (0.24)	1.60 (0.21)
Belkhiria et al. (24)	TAL	Temporal inferior	5.52 (0.26)	5.51 (0.19)
		Temporal middle	5.31 (0.21)	5.32 (0.17)
		Temporal superior	5.30 (0.25)	5.33 (0.25)
		Posterior cingulate	4.97 (0.25)	4.99 (0.18)
		Parahippocampus	5.33 (0.48)	5.36 (0.58)
Lin et al. (22)	MNI	Temporal lobe	114.80 (10.93)	114.20 (10.36)
**Parietal lobe**				
Belkhiria et al. (24)	TAL	Postcentral gyrus	3.99 (0.18)	4.08 (0.21)
Lin et al. (22)	MNI	Parietal lobe	86.1 (9.47)	85.5 (9.26)

*The original region names provided by the studies were maintained. Belkhiria et al. (66) separated region data into left and right hemisphere data (LH and RH, respectively). This separation was maintained in analysis. The insula was included in the temporal lobe because although it is covered by both the frontal and temporal lobes (68), it connects strongly to cortical and subcortical structures in the temporal lobe, e.g., the superior temporal sulcus and limbic structures (69). The fusiform gyrus data, reported by Belkhiria et al. (66), were included in both the temporal and occipital lobes, because the gyrus spans across the basal surface of both lobes. Due to a lack of reported coordinates, the regions were allocated to lobes using their verbal labels, commonly accepted allocations, and a neuroanatomy textbook (63)*.

**Table 7:** Mean analysis results for whole-brain data.

**Study**	**Hedges' *g***	**Standard error**	***z*-value**	***p*-value**	**Confidence interval**	
					**Low**	**High**
Chen et al. (64)	−0.31	0.30	−1.04	0.30	−0.90	0.28
Lin et al. (22)	0.12	0.18	0.66	0.51	−0.24	0.48
Xing et al. (65)	0.43	0.23	1.90	0.06	−0.01	0.87
Overall effect	0.12	0.19	0.64	0.52	−0.24	0.48

**Table 8:** Mean analysis results for lobe-wise data.

**Study**	**Region**	**Hedges' *g***	**Standard error**	***z*-value**	***p*-value**	**CI**	
						**Low**	**High**
**Frontal lobe**							
Belkhiria et al. (66)	LH Anterior cingulate	−0.03	0.19	−0.14	0.89	−0.40	0.35
	RH Anterior cingulate	0.13	0.19	0.67	0.50	−0.25	0.50
	LH Orbitofrontal	−0.16	0.19	−0.84	0.40	−0.53	0.21
	RH Orbitofrontal	0.09	0.19	−0.48	0.63	−0.46	0.28
Belkhiria et al. (24)	Frontal superior	0.00	0.25	0.00	>0.99	−0.49	0.49
	Anterior cingulate	−0.10	0.25	−0.40	0.69	−0.59	0.39
	Precentral gyrus	−0.29	0.25	−1.15	0.25	−0.78	0.21
Lin et al. (22)	Frontal lobe	0.13	0.18	0.69	0.49	−0.23	0.48
Overall effect		−0.03	0.07	−0.47	0.64	−0.18	0.11
**Temporal lobe**							
Belkhiria et al. (66)	LH Superior temporal	0.20	0.19	1.07	0.28	−0.17	0.58
	RH Superior temporal	−0.12	0.19	−0.62	0.53	−0.49	0.25
	LH Transverse temporal	−0.12	0.19	−0.63	0.53	−0.49	0.25
	RH Transverse temporal	−0.23	0.19	−1.20	0.23	−0.60	0.14
	LH Middle temporal	−0.01	0.19	−0.04	0.97	−0.38	0.37
	RH Middle temporal	−0.13	0.19	−0.70	0.48	−0.51	0.24
	LH Fusiform gyrus	−0.13	0.19	−1.64	0.10	−0.69	0.06
	RH Fusiform gyrus	−0.01	0.19	−0.05	0.96	−0.38	0.36
	LH Posterior cingulate	0.04	0.19	0.23	0.81	−0.33	0.42
	RH Posterior cingulate	0.00	0.19	0.00	>0.99	−0.37	0.37
	LH Insula	0.02	0.19	0.09	0.93	−0.36	0.39
	RH Insula	0.00	0.19	0.00	>0.99	−0.37	0.37
	LH Hippocampus	−0.38	0.19	−1.20	0.05	−0.76	−0.01
	RH Hippocampus	−0.55	0.19	2.84	0.01	−0.93	−0.17
	LH Amygdala	−0.37	0.19	−1.93	0.05	−0.75	0.01
	RH Amygdala	−0.26	0.19	−1.39	0.17	−0.64	0.11
Belkhiria et al. (24)	Temporal inferior	0.04	0.25	0.17	0.86	−0.45	0.53
	Temporal middle	−0.05	0.25	−0.21	0.84	−0.54	0.44
	Temporal superior	−0.12	0.25	−0.47	0.64	−0.61	0.37
	Posterior cingulate	0.09	0.25	−0.36	0.72	−0.58	0.40
	Parahippocampus	−0.06	0.25	−0.22	0.82	−0.55	0.43
Lin et al. (22)	Temporal lobe	0.06	0.18	0.31	0.76	−0.30	0.41
Overall effect		−0.12	0.04	−2.70	0.01	−0.20	−0.03
**Parietal lobe**							
Belkhiria et al. (24)	Postcentral gyrus	−0.46	0.25	−1.80	0.07	−0.95	0.04
Lin et al. (22)	Parietal lobe	0.06	0.18	0.35	0.73	−0.29	0.42
Overall effect		−0.17	0.26	−0.64	0.52	−0.67	0.34
**Occipital lobe**							
Belkhiria et al. (66)	LH Fusiform gyrus	−0.31	0.19	−1.64	0.10	−0.69	0.06
	RH Fusiform gyrus	−0.01	0.19	−0.05	0.96	−0.38	0.36
	LH Lingual gyrus	−0.20	0.19	−1.05	0.29	−0.57	0.17
	RH Lingual gyrus	0.06	0.19	0.29	0.77	−0.32	0.43
Lin et al. (22)	Occipital lobe	−0.15	0.18	−0.84	0.40	−0.51	0.20
Overall effect		−0.12	0.08	−1.47	0.14	−0.29	0.04

*LH and RH refer to left and right hemisphere, respectively. The insula was included in the temporal lobe, and the fusiform gyrus was included in both the occipital and temporal lobes (see note in Table 6 for explanations)*.

## Discussion

This meta-analysis sought to collate and evaluate the existing evidence for a difference in brain volume, specifically GM volume, in adults (aged ≥60 years) with ARHL, compared to those without ARHL. We sought to include data from both cross-sectional and longitudinal study designs, in order to consolidate and analyse the available empirical evidence and provide a better understanding of cortical changes associated with ARHL. We employed ES-SDM software to conduct analysis of neuroanatomical data across the included studies (52).

Three studies, two of which took a cross-sectional approach and one of which took a longitudinal approach, which reported GM volumes for the whole brain were included in the analysis of global neuroanatomical changes associated with ARHL. This analysis served to investigate whether the entire brain displays significant GM atrophy in individuals with ARHL, compared to those without ARHL, in order to further understand how hearing loss contributes to brain aging. The findings did not support our hypothesis that adults with ARHL would display significantly decreased whole brain GM volume, compared to those without ARHL. While previous research suggests that cross-cortical and brain wide changes are associated with ARHL (10, 22), this meta-analysis of collated studies suggests that changes associated with ARHL are not significantly greater than changes which occur in aging. If this is the case, then it is possible, as suggested by the common cause hypothesis, that a neurodegenerative factor may underly the brain atrophies observed in both hearing loss and aging.

Elevated tau protein levels could be an indicator of a potential third factor that no meta-analyzed study has accounted for explicitly. Tau is a protein found to aggregate abnormally in Alzheimer's Disease and has, therefore, been considered as a viable biomarker (70). In a study on people with dementia, the prevalence of tau protein in the cerebral spinal fluid was found to be higher in participants who reported having hearing loss than in those who did not (71). Consequently, there may be an association between tau levels and hearing. Whilst this requires further investigation, it is possible that neuroanatomical findings in the meta-analyzed studies might be influenced by biomarkers of potential pre-clinical cognitive declines (such as tau levels) in participants with ARHL.

However, limiting neuroanatomical observations to whole brain analysis only may result in overlooking of essential information regarding lobe-wise cortical changes. Understanding in which cortical structures changes occur is important for establishing the role of potential underlying causal mechanisms. Three studies, two of which took a cross-sectional approach and one of which took a longitudinal approach, which reported GM volumes in specific brain areas were included in the lobe-wise analysis of GM volumes. This analysis enabled the investigation into cortical changes across brain lobes to establish whether GM atrophy extends beyond auditory cortex (situated in temporal lobe) in individuals with ARHL. The findings support our hypothesis that decreases in GM volume observed in individuals with ARHL compared to those without ARHL occur in the temporal lobe. This is consistent with existing literature which reports increased neural atrophy in auditory cortex in individuals with ARHL, compared to those without ARHL (22). No evidence was found that declines in GM volume in people with ARHL occur in other lobes.

Research suggests that ARHL leads to up-regulation across brain networks to support speech perception, and there is evidence from functional imaging studies to support this, showing that ARHL is associated with increased functional connectivity between auditory cortex and cognitive networks (26). Increased use of such cortical resources has been theorized to trigger neurodegeneration due to over-use of neural resources and excitotoxic cell death. Yet, our data provide no evidence for declines in GM volume beyond temporal lobe and thus do not support the hypothesis that potential compensatory activity leads to neurodegeneration. This has implications for interpretation of the causal hypotheses underlying the association between hearing loss and cognitive decline. Importantly, previous research finds that declines in cognitive functioning are also associated with greater GM volume loss in temporal regions (72). This has important implications as temporal atrophies may be an underlying mechanism in the relation between hearing loss and cognitive declines.

An additional explanation for the relation between hearing loss and cognitive declines in aging not captured by this review, is the role of the psychosocial pathway in sensory deprivation: Hearing loss does not manifest exclusively in auditory deprivation due to poor hearing, but is also accompanied by mental health and well-being consequences. Adults with hearing loss may be more likely to withdraw from social interactions due to hearing difficulties, leading to experiences of increased depression, and loneliness or isolation (73). Some authors suggest that social withdrawal may exacerbate the relation between hearing loss and wider brain and cognitive health, because it increases sensory deprivation (74). As such, there may be consequences for neural and cognitive functioning if these brain areas are less utilized for stimulating social communication.

It is important to consider, with regards to both the whole-brain and lobe-wise analyses, that the included study designs varied between cross-sectional and longitudinal. First, it is possible that global GM atrophy, or atrophy across wider cortices, only occurs after prolonged sensory deprivation. In two previous longitudinal studies, a significant association between pure-tone hearing loss and reduced GM in auditory cortex was only present after at least 5 years (22, 23). Hence, it is possible theoretically that atrophies extending further than auditory cortex, or temporal lobe, may only occur after prolonged up-regulation or cortical resource reallocation to assist speech perception due to ARHL. Second, in both designs, consideration of confounding factors is important, but particularly for cross-sectional research. As such, it is important to note that differences in the controlled variables across the included studies may affect the results, and create ambiguity for interpretation. By design, longitudinal research allows for increased control over individual factors which may influence data, and hence any observed neural changes are more easily interpreted as occurring due to HL, rather than aging or another underlying neurodegenerative variable.

Importantly, to ensure homogeneity across studies included in this meta-analysis, included studies were limited to those which classified hearing status using pure tone audiometry. This method is the current gold-standard in clinical audiology, but does not account well for supra-threshold hearing difficulties, i.e., difficulties in hearing sounds presented above the auditory threshold of the listener, such as the perception of speech in background noise. Consequently, this meta-analysis does not capture the impact of such difficulties, which may present before observable declines in the audiogram are evident, on neural structure. Some studies have investigated the relation between speech reception threshold (SRT), obtained using digits-in-noise tests, on neuroanatomy. Such research found that, in older adults, poorer speech perception was associated with lower GM volume, particularly in the left superior temporal gyrus (75). Further, in older participants with Alzheimer's dementia, poorer speech perception was associated with lower cortical thickness bilaterally across many cortices (76).

Further, as many studies did not report stereotactic coordinates, the data analysis options were limited to general lobe comparisons. Hence it is not possible to interpret exactly where GM atrophy occurs within the temporal lobe. Without exact cortical locations, it is difficult to draw strong conclusions regarding the underlying neural processes or systems. Additionally, all included studies employed opportunity sampling techniques. Therefore, any generalizations were limited to the targeted populations in the included studies. Importantly, these data should be interpreted with consideration of the sample size of studies included. In order to control for confounding variables and ensure heterogeneity in methods, strict inclusion criteria were used to select the studies meta-analyzed. In-turn this resulted in a smaller number of studies selected for analysis, which resulted in a smaller number of individual data points. It has been suggested that a large sample size of individuals (across the selected studies) is required for adequate power in whole-brain meta-analysis (77). For this to be possible, there is explicit need for future large-scale longitudinal research which seeks to observe the effects of age-related hearing loss on brain morphology.

In conclusion, this meta-analysis explored the evidence for a difference in GM volume, in older adults with ARHL, compared to those without ARHL. The analysis found evidence for reduced GM volume in temporal lobes in individuals with ARHL, compared to those without ARHL. There was no evidence that GM atrophies extended to frontal, parietal, or occipital lobes, nor was there evidence for whole brain GM declines in individuals with ARHL. It is possible that significant differences in GM volume are limited to the temporal lobe, because further cortical changes only occur after a critical time period of prolonged cortical resource re-allocation. However, this finding has important implications and further longitudinal research into how neural changes across the temporal lobe in people with ARHL affects wider brain health is essential.

## Data availability statement

Publicly available datasets were analyzed in this study. This data can be found here: https://osf.io/g5qcb/.

## Author contributions

KS, HN, and CP contributed to conception of the study. KS, HN, CP, and JR contributed to the design of the study. LH provided expert advice. KS, JR, KJ, and ES contributed to article screening and data extraction. KS, JR, and LH contributed to and performed the statistical analysis. KS and JR wrote the manuscript. KS, JR, HN, CP, and LH contributed to manuscript revision, read, and approved the submitted version. All authors contributed to the article and approved the submitted version.

## Funding

The research was supported by the Biotechnology and Biological Sciences Research Council (BBSRC) Funding reference: BB/S008527/1.

## Conflict of interest

The authors declare that the research was conducted in the absence of any commercial or financial relationships that could be construed as a potential conflict of interest.

## Publisher's note

All claims expressed in this article are solely those of the authors and do not necessarily represent those of their affiliated organizations, or those of the publisher, the editors and the reviewers. Any product that may be evaluated in this article, or claim that may be made by its manufacturer, is not guaranteed or endorsed by the publisher.
