# Supplementary material for: The impact of age-related hearing loss on structural neuroanatomy: A meta-analysis
Source: Front Neurol. 2022 Aug 8;13:950997. doi: 10.3389/fneur.2022.950997 (PMC9393867; doi:10.3389/fneur.2022.950997)
Supplement: Supplementary file 1 [file Table_1.DOCX]

# Supplementary Materials 1

# Search Strings for Full Literature Search employed Across Libraries

The abbreviations “TI” and “AB” stand for “title” and “abstract”, respectively. “MH”, “Mesh” and “DE” are database specific abbreviations for indexed terms. Individual searches (S1, S2, S3) were combined to create a final search in each database (S4). Further, “+” and “Exp” indicate that the search has been exploded to include terms related to the exploded term. “*” signifies a truncated search.

| Search | The Cochrane Library | Results |
| --- | --- | --- |
| S1 | MeSH descriptor: [Hearing Loss] explode all trees OR Title (hearing NEAR/3 (loss OR impair*) OR presbycusis) OR Abstract (hearing NEAR/3 (loss OR impair*) OR presbycusis) | 3522 |
| S2 | MeSH descriptor: [Neuroanatomy] explode all trees OR MeSH descriptor: [Neuroimaging] explode all trees OR MeSH descriptor: [Anatomy] explode all trees OR MeSH descriptor: [Neurosciences] explode all trees OR MeSH descriptor: [Cerebral Cortical Thinning] explode all trees OR MeSH descriptor: [Diffusion Tensor Imaging] explode all trees OR MeSH descriptor: [Magnetic Resonance Imaging] explode all trees OR MeSH descriptor: [Gray Matter] explode all trees OR MeSH descriptor: [Brain Cortical Thickness] explode all trees OR MeSH descriptor: [White Matter] explode all trees OR Title (((“voxel based” OR voxelbased OR voxel-based NEAR/3 morphomet*) OR VBM OR ((“diffusion tensor” OR diffusion-tensor) NEAR/3 imag*) OR DTI OR ((“magnetic resonance” OR magnetic-resonance) NEAR/3 imag*) OR “cortical thickness*” OR “white matter*” OR “grey matter*” OR “gray matter*” OR “neuroimag*” OR “magnetic resonance imaging*” OR MRI) OR Abstract (((“voxel based” OR voxelbased OR voxel-based) NEAR/3 morphomet*) OR VBM OR ((“diffusion tensor” OR diffusion-tensor) NEAR/3 imag*) OR DTI OR ((“magnetic resonance” OR magnetic-resonance) NEAR/3 imag*) OR “cortical thickness*” OR “white matter*” OR “grey matter*” OR “gray matter*” OR “neuroimag*” OR “magnetic resonance imaging*” OR MRI) | 40151 |
| S3 | MeSH descriptor: [Aged] in all MeSH products OR Title (Older NEAR/3 (adult* OR man* OR men* OR woman* OR women* OR male* OR female*)) OR (elder* OR “senior citizen*” OR sexagenarian* OR septuagenarian* OR octogenarian* OR nonagenarian* OR centenarian*) OR Abstract (Older NEAR/3 (adult* OR man* OR men* OR woman* OR women* OR male* OR female*)) OR (elder* OR “senior citizen*” OR sexagenarian* OR septuagenarian* OR octogenarian* OR nonagenarian* OR centenarian*) | 261494 |
| S4 | S1 AND S2 AND S3 | 16 |
|  | MEDLINE Complete |  |
| S1 | (MH “Hearing Loss+”) OR TI (Hearing N3 (loss OR impair*) OR presbycusis) OR AB (Hearing N3 (loss OR impair*) OR presbycusis) | 98113 |
| S2 | (MH "Neuroanatomy") OR (MH “Neuroimaging+”) OR (MH "Anatomy+") OR (MH "Neurosciences+") OR (MH "Cerebral Cortical Thinning") OR (MH "Diffusion Tensor Imaging") OR (MH "Magnetic Resonance Imaging+") OR (MH "Gray Matter") OR (MH "Brain Cortical Thickness") OR (MH "White Matter") OR TI (((“voxel based” OR voxelbased OR voxel-based) N3 morphomet*) OR VBM OR ((“diffusion tensor” OR diffusion-tensor) N3 imag*) OR DTI OR ((“magnetic resonance” OR magnetic-resonance) N3 imag*) OR “cortical thickness*” OR “white matter*” OR “grey matter*” OR “gray matter*” OR neuroimag* OR “magnetic resonance imaging*” OR MRI) OR AB (((“voxel based” OR voxelbased OR voxel- based) N3 morphomet*) OR VBM OR ((“diffusion tensor” OR diffusion-tensor) N3 imag*) OR DTI OR ((“magnetic resonance” OR magnetic-resonance) N3 imag*) OR “cortical thickness*” OR “white matter*” OR “grey matter*” OR “gray matter*” OR neuroimag* OR “magnetic resonance imaging*” OR MRI) | 1207617 |
| S3 | (MH "Aged+") OR (MH "Aged, 80 and over") OR TI (Older N3 (adult* OR man* OR men* OR woman* OR women* OR male* OR female*) OR (patient* N3 (presbycusis OR PC)) OR (elder* OR “senior citizen*” OR sexagenarian* OR septuagenarian OR octogenarian OR nonagenarian* OR centenarian*) OR ((60 OR 61 OR 62 OR 63 OR 64 OR 65) N2 (year* OR old* OR aged))) OR AB (Older N3 (adult* OR man* OR men* OR woman* OR women* OR male* OR female*) OR (patient* N3 (presbycusis OR PC)) OR (elder* OR “senior citizen*” OR sexagenarian* OR septuagenarian OR octogenarian OR nonagenarian* OR centenarian*) OR ((60 OR 61 OR 62 OR 63 OR 64 OR 65) N2 (year* OR old* OR aged))) | 3483996 |
| S4 | S1 AND S2 AND S3 | 1371 |
|  | Web of Science |  |
| S1 | (TI = ((Hearing near/3 (loss OR impair*)) OR presbycusis) OR (AB = ((Hearing near/3 (loss OR impair*)) OR presbycusis) | 55801 |
| S2 | (TI = (((“voxel based” OR voxelbased OR voxel-based) near/3 morphomet*) OR VBM OR ((“diffusion tensor” OR diffusion-tensor) near/3 imag*) OR DTI OR ((“magnetic resonance” OR magnetic-resonance) near/3 imag*) OR “cortical thickness*” OR “white matter*” OR “grey matter*” OR “gray matter*” OR neuroimag* OR “magnetic resonance imaging*” OR MRI OR neuroanatom* OR anatom* OR neuroscien* OR "cerebral cortical thinning") OR AB = (((“voxel based” OR voxelbased OR voxel-based) near/3 morphomet*) OR VBM OR ((“diffusion tensor” OR diffusion-tensor) near/3 imag*) OR DTI OR ((“magnetic resonance” OR magnetic- resonance) near/3 imag*) OR “cortical thickness*” OR “white matter*” OR “grey matter*” OR “gray matter*” OR neuroimag* OR “magnetic resonance imaging*” OR MRI OR neuroanatom* OR anatom* OR neuroscien* OR "cerebral cortical thinning") | 911666 |
| S3 | (AB = ((older adult*) OR (adult*) OR (elder*) OR (senior citizen*) OR (sexagenarian*))) OR TI = (((older adult*) OR (adult*) OR (elder*) OR (senior citizen*) OR (sexagenarian*))) | 1746214 |
| S4 | S1 AND S2 AND S3 | 366 |
|  | PubMed |  |
| S1 | "Hearing Loss"[Mesh] OR (“hearing loss” [Title/Abstract] OR “hearing impair*” [Title/Abstract] OR presbycusis [Title/Abstract]) | 97136 |
| S2 | "Neuroanatomy" [MeSH Terms] OR "Neuroimaging" [MeSH Terms] OR "Anatomy" [MeSH Terms] OR "Neurosciences" [MeSH Terms] OR "Cerebral Cortical Thinning" [MeSH Terms] OR "Diffusion Tensor Imaging" [MeSH Terms] OR "Magnetic Resonance Imaging" [MeSH Terms] OR "Gray Matter" [MeSH Terms] OR "Brain Cortical Thickness" [MeSH Terms] OR "White Matter" [MeSH Terms] OR ("Voxel-based morphometry" [Title/Abstract] OR VBM [Title/Abstract] OR "diffusion tensor imaging*" [Title/Abstract] OR DTI [Title/Abstract] OR "cortical thickness*" [Title/Abstract] OR "white matter*" [Title/Abstract] OR "grey matter*" [Title/Abstract] OR “gray matter*” [Title/Abstract] OR “neuroimag*” [Title/Abstract] OR "magnetic resonance imaging*" [Title/Abstract] OR MRI [Title/Abstract]) | 1209913 |
| S3 | "Aged" [Mesh] OR "Aged, 80 and over" [Mesh] OR “adult*” [Title/Abstract] OR “patient*” [Title/Abstract] OR “older adult*” [Title/Abstract] OR “older man*” [Title/Abstract] OR “older men*” [Title/Abstract] OR “older woman*” [Title/Abstract] OR “older women*” [Title/Abstract] OR “older male*” [Title/Abstract] OR “older female*” [Title/Abstract] OR “elder*” [Title/Abstract] OR “senior citizen*” [Title/Abstract] OR “sexagenarian*” [Title/Abstract] OR “septuagenarian*” [Title/Abstract] OR “octogenarian*” [Title/Abstract] OR “nonagenarian*” [Title/Abstract] OR “centenarian*” [Title/Abstract] OR "61" [Title/Abstract] OR "62" [Title/Abstract] OR "63" [Title/Abstract] OR "64" [Title/Abstract] OR "65" [Title/Abstract] OR "66" [Title/Abstract] OR "67" [Title/Abstract] OR "68" [Title/Abstract] OR "69" [Title/Abstract] | 10332317 |
| S4 | S1 AND S2 AND S3 | 4669 |
|  | Scopus |  |
| S1 | TITLE-ABS ("hearing loss” OR (hearing W/3 loss) OR (hearing W/3 impair*) OR presbycusis) | 78977 |
| S2 | TITLE-ABS (((“voxel based” OR voxelbased OR voxel-based) W/3 morphomet*) OR ((“diffusion tensor” OR diffusion-tensor) W/3 imag*) OR ((“magnetic resonance” OR magnetic-resonance) W/3 imag*) OR “cortical thickness*” OR “white matter*” OR “grey matter*” OR “gray matter*” OR neuroimag* OR “magnetic resonance imaging*” OR “neuroanatom*” OR “neuroscien*” OR "cerebral cortical thinning") | 521901 |
| S3 | TITLE-ABS (older W/3 adult*) OR (older W/3 man*) OR (older W/3 men*) OR (older W/3 woman*) OR (older W/3 women*) OR (older W/3 male*) OR (older W/3 female*) OR (elder*) OR ("senior citizen*") OR (sexagenarian*) OR (septuagenarian*) OR (octogenarian*) OR (nonagenarian*) OR (centenarian*) | 3126310 |
| S4 | S1 AND S2 AND S3 | 871 |
|  | Embase |  |
| S1 | Exp “hearing impaired person+” OR exp “hearing impairment+” OR Title ((hearing ADJ3 (loss OR impair*)) OR presbycusis) OR Abstract ((hearing ADJ3 (loss OR impair*)) OR presbycusis) | 128431 |
| S2 | Exp neuroanatomy/ OR exp neuroimaging/ OR exp neuroscience/ OR exp "cortical thickness (brain)"/ OR exp diffusion tensor imaging/ OR magnetic resonance imaging.mp. OR exp gray matter/ OR exp white matter/ OR Title (((voxel based OR voxelbased OR voxel-based) ADJ3 morphomet*) OR Voxel-based morphometry OR VBM OR ((diffusion tensor OR diffusion- tensor) ADJ3 imag*) OR diffusion tensor imaging* OR DTI OR ((magnetic resonance OR magnetic-resonance) ADJ3 imag*) OR cortical thickness* OR white matter* OR grey matter* OR gray matter* OR neuroimag* OR magnetic resonance imaging* OR MRI) OR Abstract (((voxel based OR voxelbased OR voxel-based) ADJ3 morphomet*) OR Voxel-based morphometry OR VBM OR ((diffusion tensor OR diffusion-tensor) ADJ3 imag*) OR diffusion tensor imaging* OR DTI OR ((magnetic resonance OR magnetic-resonance) ADJ3 imag*) OR cortical thickness* OR white matter* OR grey matter* OR gray matter* OR neuroimag* OR magnetic resonance imaging* OR MRI) | 1364664 |
| S3 | Exp “aged+” OR Title (Older ADJ3 (adult* OR man* OR men* OR woman* OR women* OR male* OR female*) OR adult* OR elder* OR senior citizen* OR sexagenarian* OR septuagenarian* OR octogenarian* OR nonagenarian* OR centenarian* OR old*) OR ((presbycusis OR PC) adj3 patient*) OR Abstract (Older ADJ3 (adult* OR man* OR men* OR woman* OR women* OR male* OR female*) OR adult* OR elder* OR senior citizen* OR sexagenarian* OR septuagenarian* OR octogenarian* OR nonagenarian* OR centenarian* OR old*) OR ((presbycusis OR PC) adj3 patient*) | 6233392 |
| S4 | S1 AND S2 AND S3 | 6,434 |
|  | PsycINFO |  |
| S1 | (DE “Hearing Loss+”) OR TI (Hearing N3 (loss OR impair*) OR presbycusis) OR AB (Hearing N3 (loss OR impair*) OR presbycusis) | 24043 |
| S2 | (DE "Neuroanatomy+") OR (DE “Neuroimaging+”) OR (DE "Anatomy+") OR (DE "Neurosciences+") OR (DE "Cerebral Atrophy") OR (DE "Diffusion Tensor Imaging") OR (DE "Magnetic Resonance Imaging+") OR (DE "Gray Matter") OR (DE "White Matter+") OR TI (((“voxel based” OR voxelbased OR voxel-based) N3 morphomet*) OR VBM OR ((“diffusion tensor” OR diffusion-tensor) N3 imag*) OR DTI OR ((“magnetic resonance” OR magnetic-resonance) N3 imag*) OR “cortical thickness*” OR “white matter*” OR “grey matter*” OR “gray matter*” OR “neuroimag*” OR “magnetic resonance imaging*” OR MRI) OR AB (((“voxel based” OR voxelbased OR voxel-based) N3 morphomet*) OR VBM OR ((“diffusion tensor” OR diffusion-tensor) N3 imag*) OR DTI OR ((“magnetic resonance” OR magnetic-resonance) N3 imag*) OR “cortical thickness*” OR “white matter*” OR “grey matter*” OR “gray matter*” OR “neuroimag*” OR “magnetic resonance imaging*” OR MRI) | 267492 |
| S3 | (DE "Aged+") OR (MH "Aged, 80 and over") OR TI (Older N3 (adult* OR man* OR men* OR woman* OR women* OR male* OR female*) OR (elder* OR “senior citizen*” OR sexagenarian* OR septuagenarian OR octogenarian OR nonagenarian* OR centenarian*) OR ((60 OR 61 OR 62 OR 63 OR 64 OR 65) N2 (year* OR old* OR aged))) OR AB (Older N3(adult* OR man* OR men* OR woman* OR women* OR male* OR female*) OR (elder* OR “senior citizen*” OR sexagenarian* OR septuagenarian OR octogenarian OR nonagenarian* OR centenarian*) OR ((60 OR 61 OR 62 OR 63 OR 64 OR 65) N2 (year* OR old* OR aged))) | 155573 |
| S4 | S1 AND S2 AND S3 | 63 |
|  | CINAHL |  |
| S1 | (DE "Hearing Loss, Functional") OR TI (Hearing N3 (loss OR impair*) OR presbycusis) OR AB (Hearing N3 (loss OR impair*) OR presbycusis) | 21936 |
| S2 | (DE "Neuroanatomy") OR (DE “Neuroimaging”) OR (DE "Anatomy+") OR (DE "Neurosciences+") OR (DE "Cerebral Cortical Thinning") OR (DE "Magnetic Resonance Imaging+") OR (DE "Gray Matter") OR (DE "Brain Cortical Thickness") OR (DE "White Matter") OR TI (((“voxel based” OR voxelbased OR voxel-based) N3 morphomet*) OR VBM OR ((“diffusion tensor” OR diffusion-tensor) N3 imag*) OR DTI OR ((“magnetic resonance” OR magnetic-resonance) N3 imag*) OR “cortical thickness*” OR “white matter*” OR “grey matter*” OR “gray matter*” OR “neuroimag*” OR “magnetic resonance imaging*” OR MRI) OR AB (((“voxel based” OR voxelbased OR voxel-based) N3 morphomet*) OR VBM OR ((“diffusion tensor” OR diffusion-tensor) N3 imag*) OR DTI OR ((“magnetic resonance” OR magnetic-resonance) N3 imag*) OR “cortical thickness*” OR “white matter*” OR “grey matter*” OR “gray matter*” OR “neuroimag*” OR “magnetic resonance imaging*” OR MRI) | 190594 |
| S3 | (DE "Aged+") OR (MH "Aged, 80 and over+") OR TI (Older N3 (adult* OR man* OR men* OR woman* OR women* OR male* OR female*) OR (elder* OR “senior citizen*” OR sexagenarian* OR septuagenarian OR octogenarian OR nonagenarian* OR centenarian*) OR ((60 OR 61 OR 62 OR 63 OR 64 OR 65) N2 (year* OR old* OR aged))) OR AB (Older N3 (adult* OR man* OR men* OR woman* OR women* OR male* OR female*) OR (elder* OR “senior citizen*” OR sexagenarian* OR septuagenarian OR octogenarian OR nonagenarian* OR centenarian*) OR ((60 OR 61 OR 62 OR 63 OR 64 OR 65) N2 (year* OR old* OR aged))) | 952171 |
| S4 | S1 AND S2 AND S3 | 244 |
|  | Academic Search Ultimate |  |
| S1 | DE “HEARING impaired+” OR TI (hearing N3 (loss OR impair*) OR presbycusis) OR AB (hearing N3 (loss OR impair*) OR presbycusis) | 36394 |
| S2 | DE “Neuroanatomy+” OR DE “Brain imaging+” OR DE “anatomy+” OR DE “neurosciences+” OR DE “diffusion tensor imaging” OR DE “magnetic resonance imaging+” OR DE “gray matter (nerve tissue)” OR DE “white matter (nerve tissue)+” OR TI (((“voxel based” OR voxelbased OR voxel-based) N3 morphomet*) OR VBM OR ((“diffusion tensor” OR diffusion-tensor) N3 imag*) OR DTI OR ((“magnetic resonance” OR magnetic-resonance) N3 imag*) OR “cortical thickness*” OR “white matter*” OR “grey matter*” OR “gray matter*” OR “neuroimag*” OR “magnetic resonance imaging*” OR MRI OR neuroanatom* OR anatom* OR neuroscien* OR "cerebral cortical thinning") OR AB (((“voxel based” OR voxelbased OR voxel-based) N3 morphomet*) OR VBM OR ((“diffusion tensor” OR diffusion-tensor) N3 imag*) OR DTI OR ((“magnetic resonance” OR magnetic-resonance) N3 imag*) OR “cortical thickness*” OR “white matter*” OR “grey matter*” OR “gray matter*” OR “neuroimag*” OR “magnetic resonance imaging*” OR MRI OR neuroanatom* OR anatom* OR neuroscien* OR "cerebral cortical thinning") | 707936 |
| S3 | DE "OLDER people+" OR TI ( (Older N3 (adult* OR man* OR men* OR woman* OR women* OR male* OR female*)) OR (elder* OR “senior citizen*” OR sexagenarian* OR septuagenarian* OR octogenarian* OR nonagenarian* OR centenarian*) OR AB ( (Older N3 (adult* OR man* OR men* OR woman* OR women* OR male* OR female*) OR (elder* OR “senior citizen*” OR sexagenarian* OR septuagenarian* OR octogenarian* OR nonagenarian* OR centenarian*) | 297950 |
| S4 | S1 AND S2 AND S3 | 88 |
